# Supplementary material for: DIPLI: deep image prior lucky imaging for blind astronomical image restoration
Source: Sci Rep. 2026 Apr 15;16:17204. doi: 10.1038/s41598-026-47300-4 (PMC13234401; doi:10.1038/s41598-026-47300-4)
Supplement: Supplementary file 1 — Supplementary Information. [file 41598_2026_47300_MOESM1_ESM.pdf]

Supplementary Information

DIPLI: Deep Image Prior Lucky Imaging for Blind Astronomical Image Restoration

Suraj Singh[1,*,†], Anastasiia Batsheva[1,2,†], Oleg Y. Rogov[1,2], Ahmed Bouridane[3]

[1]Skolkovo Institute of Science and Technology, Moscow, Russia
[2]AIRI, Moscow, Russia
[3]University of Sharjah, UAE
[†]Contributed equally. [*]Corresponding author: Suraj.Singh@skoltech.ru

---

Contents

1 Supplementary Note 1: Confidence Weighting Ablation **1**
1.1 Protocol . . . 1
1.2 Results and discussion . . . 2

2 Supplementary Note 2: SGLD Convergence Diagnostics **2**
2.1 Protocol . . . 2
2.2 Results and discussion . . . 3

3 Supplementary Note 3: Extended Frame Count Ablation **5**
3.1 Protocol . . . 5
3.2 Results and discussion . . . 5

4 Supplementary Note 4: Failure Case Analysis **6**
4.1 Protocol . . . 7
4.2 Results and discussion . . . 7

5 Supplementary Note 5: Real Astronomical Data: ZTF Reconstructions **8**
5.1 Data description . . . 8
5.2 Results and discussion . . . 8

---

1 Supplementary Note 1: Confidence Weighting Ablation

To validate the confidence-weighted back-projection loss (Eq. 11 in the main text), we performed a controlled ablation across all 12 synthetic benchmark scenes, comparing $\alpha = 0$ (confidence OFF, i.e., uniform weighting across all pixels) against $\alpha = 1$ (confidence ON, i.e., flow-gradient-based weighting as described in Section 3 of the main text).

1.1 Protocol

Each scene was processed twice under identical hyperparameters ($K = 11$, $N = 6500$, $n_0 = 6000$, $\sigma_\xi = 0.0025$, $n_{\text{channels}} = 128$, seed = 42), differing only in α. The confidence map entering the loss function is computed as:

$$c_k(p) = \exp\big(-\alpha \, \|\nabla \omega_k(p)\|_F\big),$$

Setting $\alpha = 0$ yields $c_k(p) = 1$ everywhere (uniform weighting), while $\alpha = 1$ activates the full confidence map.

1.2 Results and discussion

	Confidence		α = 0 (OFF)				α = 1 (ON)			
Scene	$\bar{c}_k^{\alpha=0}$	$\bar{c}_k^{\alpha=1}$	PSNR	SSIM	LPIPS	DISTS	PSNR	SSIM	LPIPS	DISTS
01	0.932	0.921	21.94	0.514	0.106	0.083	**21.95**	**0.517**	**0.091**	**0.069**
02	0.960	0.956	30.42	0.505	**0.060**	**0.096**	**30.45**	**0.507**	**0.060**	0.099
03	0.958	0.957	**23.84**	**0.327**	**0.094**	**0.067**	23.83	0.325	0.099	0.078
04	0.955	0.951	**30.36**	**0.732**	**0.088**	0.107	**30.36**	**0.732**	**0.088**	**0.106**
05	0.951	0.948	**28.57**	**0.725**	**0.124**	**0.116**	28.50	0.723	0.127	0.121
06	0.954	0.955	**26.62**	**0.422**	**0.100**	**0.092**	26.59	0.421	0.108	0.103
07	0.958	0.957	**25.94**	**0.375**	**0.080**	**0.068**	25.88	0.372	0.087	0.077
08	0.940	0.944	**22.44**	**0.515**	**0.138**	**0.127**	22.43	0.514	0.140	0.129
09	0.953	0.957	**24.52**	**0.317**	**0.099**	**0.094**	24.50	0.315	0.106	0.101
10	0.954	0.953	**26.11**	**0.666**	**0.144**	**0.151**	26.07	**0.666**	0.145	0.152
11	0.955	0.955	**25.70**	**0.728**	**0.123**	**0.140**	25.67	0.724	0.136	0.149
12	0.954	0.954	**26.04**	**0.634**	**0.158**	0.147	**26.04**	0.633	0.160	**0.146**
**Mean $\|\Delta\|$:**			PSNR: 0.03 dB, SSIM: 0.002				LPIPS: 0.006, DISTS: 0.005			

Supplementary Table 1: Full confidence weighting ablation results. $\bar{c}_k$ denotes mean flow confidence. For LPIPS and DISTS, lower is better; for PSNR and SSIM, higher is better.

The results indicate that confidence weighting acts as a **safety mechanism** rather than a universal performance booster:

1. **Neutral on well-registered data.** All 12 benchmark scenes exhibit high mean confidence ($\bar{c}_k > 0.93$), so the confidence maps are near-uniform. The weighting correctly determines that no down-weighting is needed, yielding negligible metric changes (mean $|\Delta\text{PSNR}| < 0.1$ dB, mean $|\Delta\text{LPIPS}| < 0.01$).
2. **Proportional engagement with flow unreliability.** Scene 01, the scene with the lowest mean confidence ($\bar{c}_k = 0.932$), exhibits the largest benefit: LPIPS improves by 14% (0.106 → 0.091) and DISTS by 16% (0.083 → 0.069).
3. **No systematic degradation.** The negligible differences on well-registered scenes confirm that confidence weighting does not introduce harmful bias when the optical flow is reliable.

A further demonstration of the confidence map under degraded registration is provided in Supplementary Note 4 (Failure Case Analysis), where the star field's lower confidence correctly flags unreliable flow. On such poorly registered data, the confidence map down-weights the most erroneous pixels. However, because the star field failure also involves a fundamental prior mismatch (see Supplementary Note 4), confidence weighting alone is insufficient to recover the reconstruction.

2 Supplementary Note 2: SGLD Convergence Diagnostics

Cheng et al. [4] established theoretical convergence guarantees for SGLD in the single-frame DIP setting. DIPLI extends this framework to a multi-frame back-projection loss that aggregates K frames into a single objective. Although the SGLD update rule retains the same form (Algorithm 1 in the main text), the loss landscape geometry changes due to the multi-frame aggregation. Because formal convergence analysis of SGLD under multi-frame aggregation with mini-batch sampling has not yet been established, we provide empirical convergence evidence through a multi-chain analysis using the Gelman–Rubin $\hat{R}$ statistic [1] and effective sample size (ESS).

2.1 Protocol

Three independent SGLD chains were run with different random seeds on each of three representative scenes (Scene 01: grayscale; Scene 06: RGB; Scene 10: RGB). All chains shared the same hyperparameters ($K = 11$, $N = 6500$, $n_0 = 6000$, $\sigma_\xi = 0.0025$) and retained the last 500 posterior samples (i.e., MCMC iterates of the network output).

The Gelman–Rubin $\hat{R}$ statistic was computed per pixel across the three chains, with $\hat{R} < 1.1$ serving as the standard convergence threshold [1]. Effective sample size (ESS) was estimated via the initial positive sequence estimator; ESS > 100 is generally considered adequate for posterior mean estimation.

Latent perturbation scale $\sigma_z = 0.01$**.** In addition to SGLD parameter noise, a small perturbation is applied to the input latent code z at each iteration (Eq. 15 in the main text). Cheng et al. [4] reported limited benefit from latent perturbation alone in single-frame Bayesian DIP. We retain it following standard practice, as it incurs negligible computational overhead (<1% wall-clock time).

2.2 Results and discussion

Supplementary Table 2: SGLD convergence diagnostics across three scenes and three independent chains.

Scene	Median $\hat{R}$	% $\hat{R} < 1.1$	% $\hat{R} < 1.05$	Mean ESS	% ESS > 100
01 (gray)	1.100	50.1%	29.7%	253	89.9%
06 (RGB)	1.010	86.8%	74.7%	172	97.9%
10 (RGB)	1.059	66.0%	45.3%	205	86.0%

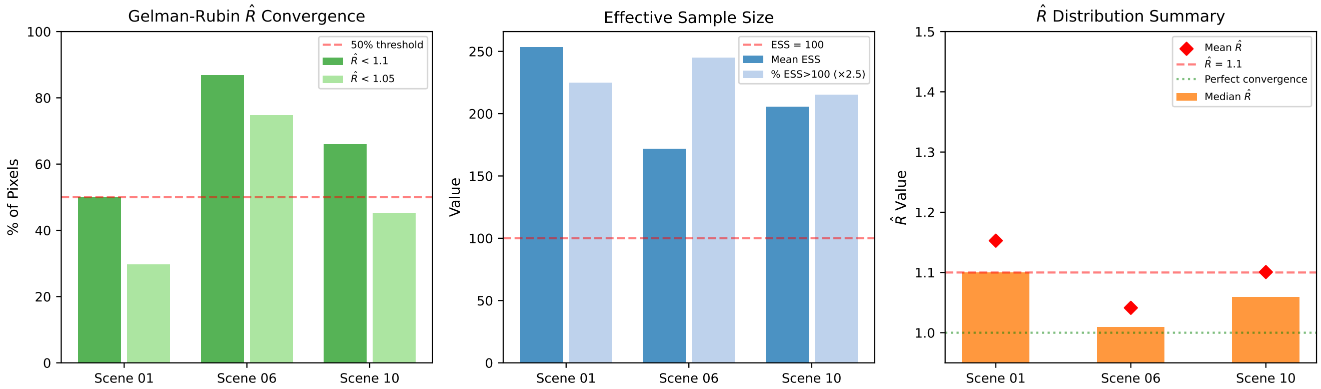

Supplementary Figure 1: Summary of SGLD convergence diagnostics. **Left:** Percentage of pixels satisfying $\hat{R} < 1.1$ and $\hat{R} < 1.05$. **Center:** Mean effective sample size and percentage above 100. **Right:** Median and mean $\hat{R}$ values relative to the 1.1 threshold.

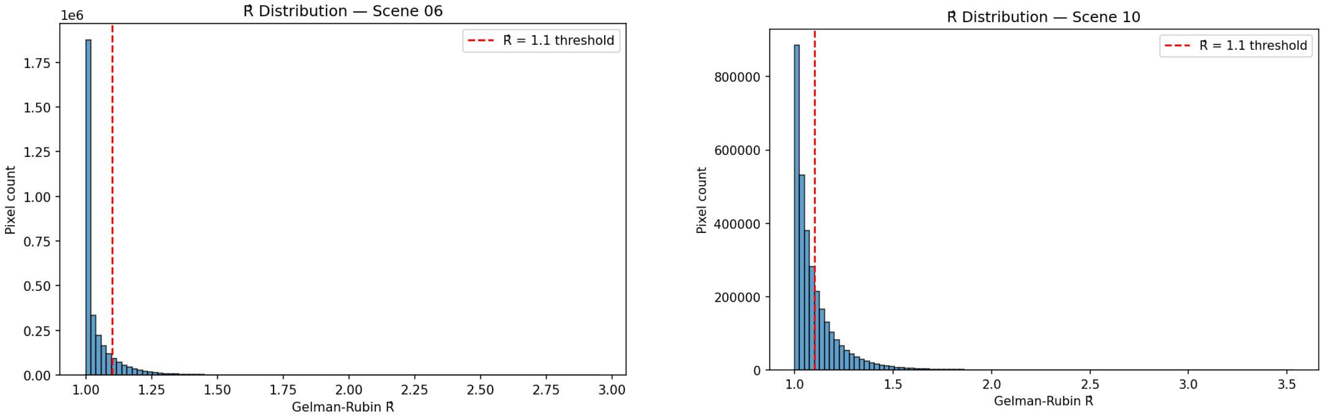

Supplementary Figure 2: $\hat{R}$ histograms for Scene 06 (left) and Scene 10 (right). The red dashed line marks the $\hat{R} = 1.1$ convergence threshold. The bulk of pixels are concentrated near $\hat{R} = 1.0$, indicating good convergence for the majority of the image.

The results demonstrate **approximate convergence** sufficient for posterior mean estimation, with scene-dependent mixing quality:

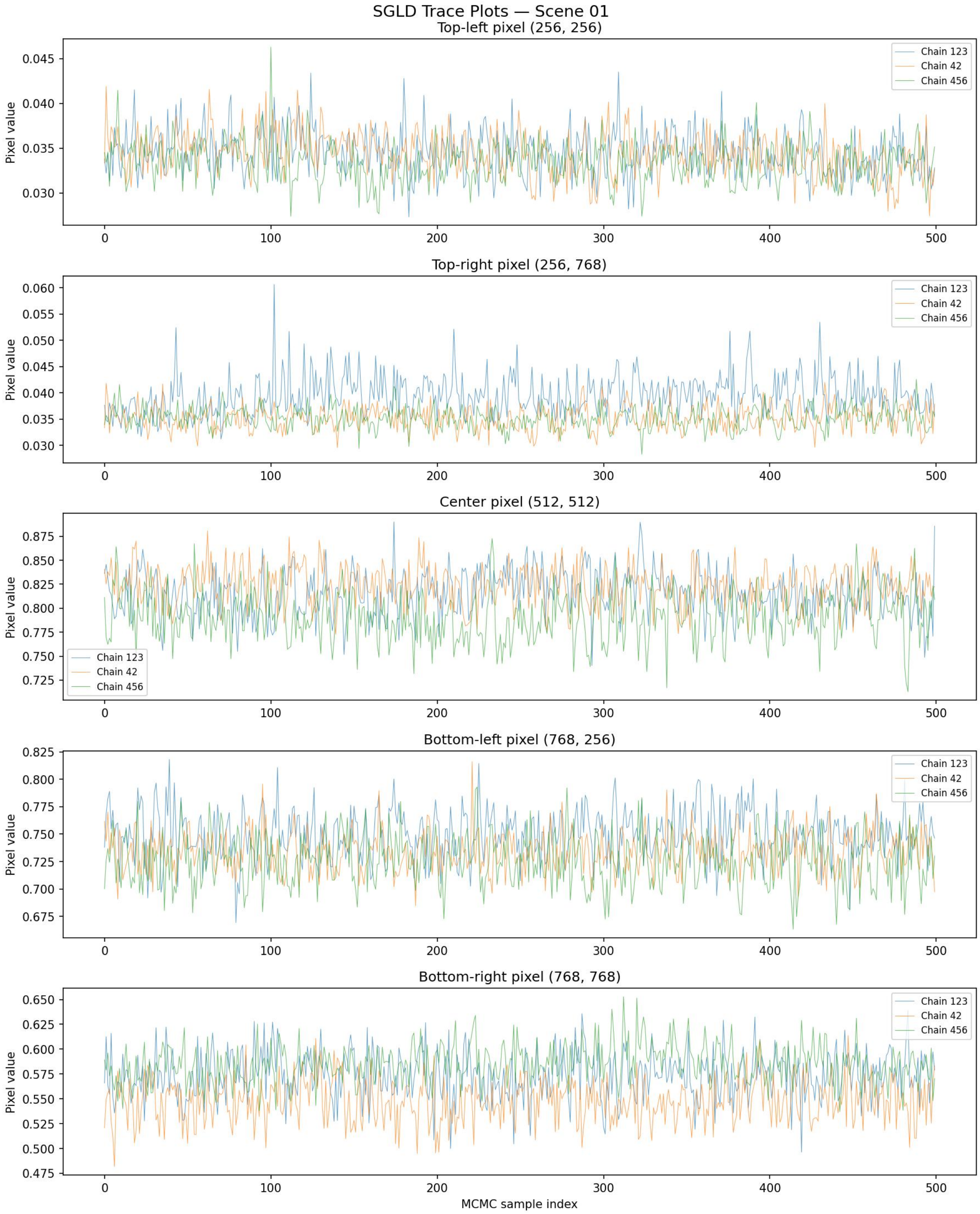

Supplementary Figure 3: SGLD trace plots for Scene 01 at five representative pixel locations. Three chains (seeds 42, 123, 456) are shown in different colors. The chains track similar stationary distributions without visible drift, supporting approximate convergence.

- **Scene 06** shows excellent convergence: 86.8% of pixels satisfy $\hat{R} < 1.1$, and 97.9% have ESS > 100.
- **Scene 10** shows good convergence: 66.0% of pixels fall below $\hat{R} = 1.1$, with a high mean ESS of 205.

- **Scene 01** (grayscale) shows the weakest convergence: only 50.1% of pixels satisfy $\hat{R} < 1.1$, well below the conventional benchmark for full convergence (>95% of pixels below 1.1). However, the ESS for Scene 01 is the highest of all three scenes (mean 253, with 89.9% above 100), indicating that individual chains explore the posterior efficiently despite weaker inter-chain agreement. This discrepancy suggests that the three chains settle into slightly different posterior modes in some pixel regions, while still producing similar aggregate statistics.
- **RGB vs. grayscale**: RGB scenes converge more quickly than the grayscale scene, likely due to the richer gradient signal provided by three color channels.

The trace plots in Supplementary Figure 3 visually confirm that the three chains track similar stationary distributions without systematic drift, supporting approximate convergence even where $\hat{R}$ is elevated.

Achieving strict convergence (>95% of pixels below $\hat{R} = 1.1$) would require longer sampling chains or an annealed noise schedule, increasing runtime by up to 20×. For the posterior *mean* estimation that DIPLI targets, however, the current convergence level is adequate for two reasons.

First, 86–98% of pixels have ESS > 100, which bounds the Monte Carlo standard error of the posterior mean to $\lesssim 10\%$ of the posterior standard deviation per pixel [1]. Second, the U-Net generator enforces strong spatial correlation between neighboring pixels through its convolutional structure and skip connections. As a result, the posterior mean at any given pixel is effectively regularized by its neighbors, even when its own chain mixes slowly.

Empirically, reconstruction quality (PSNR and LPIPS relative to ground truth) remains stable across seeds, as confirmed by the multi-seed experiments in this section and in Supplementary Note 3.

3 Supplementary Note 3: Extended Frame Count Ablation

To confirm that the $K \in [7, 13]$ operating range identified in the main text is robust across scenes, rather than an artifact of a single evaluation condition, we performed an extended frame-count ablation.

3.1 Protocol

Four representative scenes (01 and 03: grayscale; 06 and 10: RGB) were each processed with $K \in \{1, 3, 5, 7, 11, 15, 25, 50\}$ input frames, yielding 32 runs in total. All other hyperparameters were held fixed ($\alpha = 1.0$, $N = 6500$, $n_0 = 6000$, $\sigma_\xi = 0.0025$, seed = 42). Because each condition uses a single seed, individual data points carry inherent stochastic variance from SGLD sampling and mini-batch selection. The analysis therefore focuses on robust trends (plateau shape and monotonic regions) rather than individual point estimates.

Mini-batch size K_b. At each optimization step, K_b frames are randomly sampled from the K available inputs to compute the loss gradient. We set $K_b = 4$ as a practical trade-off: lower K_b increases gradient variance, while higher K_b raises per-iteration cost. With $K = 11$ and $K_b = 4$, each frame appears in approximately 36% of updates, providing sufficient coverage while maintaining stochastic diversity. The consistent performance plateau across $K \in [7, 13]$ suggests that the pipeline is not sensitive to the exact fraction of frames seen per step.

3.2 Results and discussion

Across all scenes, performance follows a consistent three-stage pattern: (1) a *rapid improvement* phase ($K < 5$), where each additional frame contributes substantial new information; (2) a *plateau* ($K \in [5, 15]$), where gains become marginal and performance stabilizes; and (3) *gradual degradation* ($K > 25$), where accumulated registration errors from additional frames begin to erode reconstruction quality.

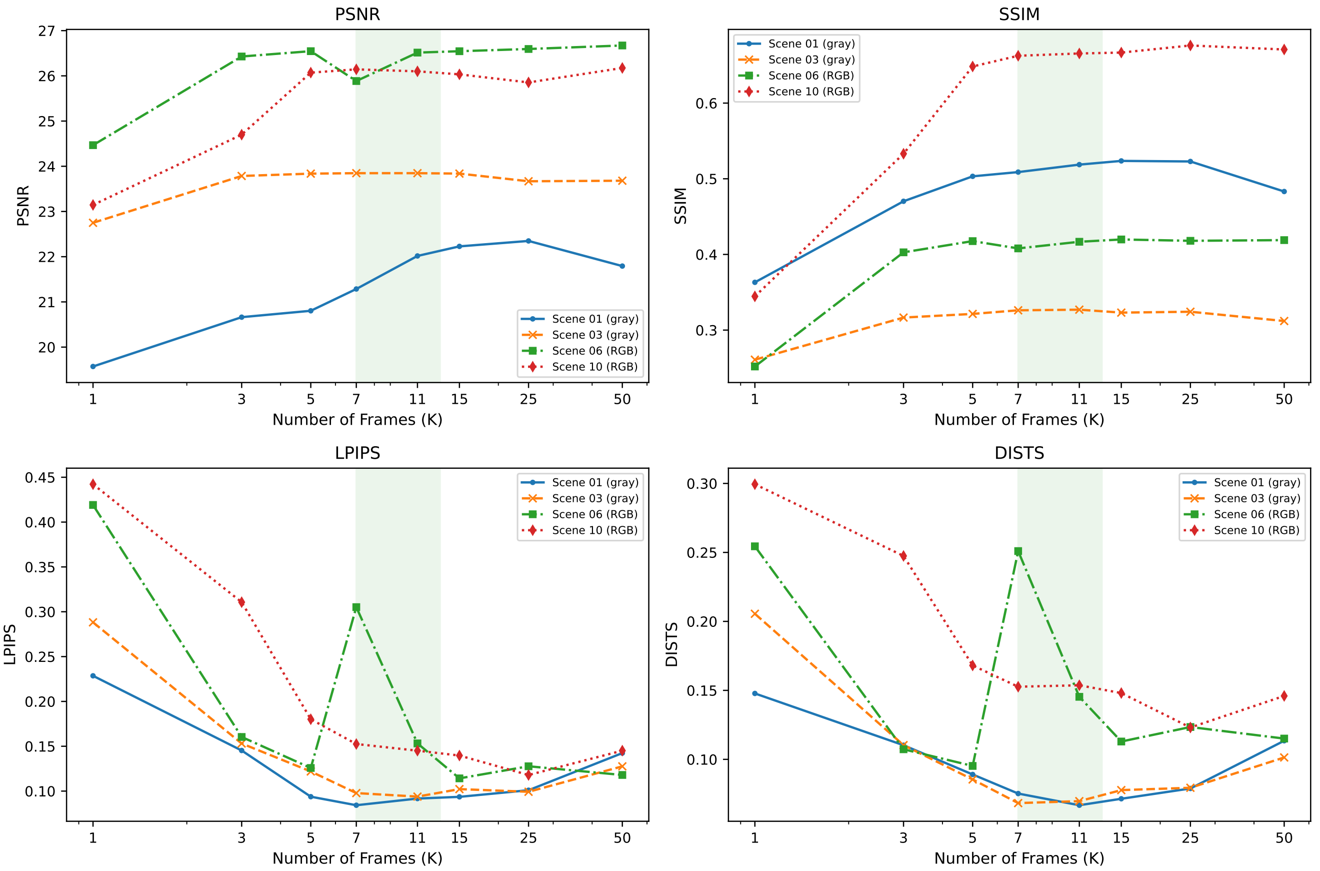

Supplementary Figure 4: Frame count ablation across 4 scenes and 4 metrics. The green shaded region marks $K = [7, 13]$. All scenes show a consistent plateau-then-decline pattern, with the perceptual optimum (LPIPS, DISTS) falling within the $K = [7, 13]$ range.

	$K=1$	$K=3$	$K=5$	$K=7$	$K=11$	$K=15$	$K=25$	$K=50$
				PSNR (dB) ↑				
Scene 01	19.57	20.66	20.80	21.29	22.02	22.23	**22.35**	21.79
Scene 03	22.75	23.79	23.84	23.85	**23.85**	23.84	23.67	23.68
Scene 06	24.47	26.43	26.55	25.89	26.51	26.54	26.59	**26.67**
Scene 10	23.14	24.70	26.07	26.14	26.10	26.03	25.85	**26.17**
				LPIPS ↓				
Scene 01	0.229	0.145	0.094	**0.084**	0.092	0.094	0.101	0.142
Scene 03	0.288	0.153	0.122	**0.098**	0.094	0.102	0.099	0.128
Scene 06	0.419	0.160	0.126	0.305*	0.153	**0.114**	0.128	0.118
Scene 10	0.442	0.311	0.180	0.152	0.145	0.140	**0.118**	0.145

*Scene 06, $K=7$: outlier attributable to single-seed stochastic variance (cf. $K=5$: 0.126, $K=11$: 0.153).

Supplementary Table 3: PSNR and LPIPS values across frame counts K for four scenes. Bold indicates the best value per scene.

4 Supplementary Note 4: Failure Case Analysis

To probe the boundaries of DIPLI's applicability, we designed a controlled experiment comparing performance on a synthetic star field (composed of point-source objects) against a resolved scene (Scene 03, Moon surface).

4.1 Protocol

Three runs were performed:

1. **Star field + TVNet**: A synthetic star field with 150 point sources (Gaussian PSFs, $\sigma = 1.5$ px), degraded with the same pipeline as the benchmark scenes, processed with TVNet flow estimation.

2. **Star field + TV-L1**: The same star field processed with classical TV-L1 optical flow instead of TVNet, to determine whether the failure is specific to the learned flow estimator.

3. **Resolved Scene 03 + TVNet**: A resolved-object scene (Moon surface) serving as a control, demonstrating successful reconstruction under identical hyperparameters.

All runs used $K = 11$, $\alpha = 1.0$, $N = 6500$, $\times 4$ upscaling.

4.2 Results and discussion

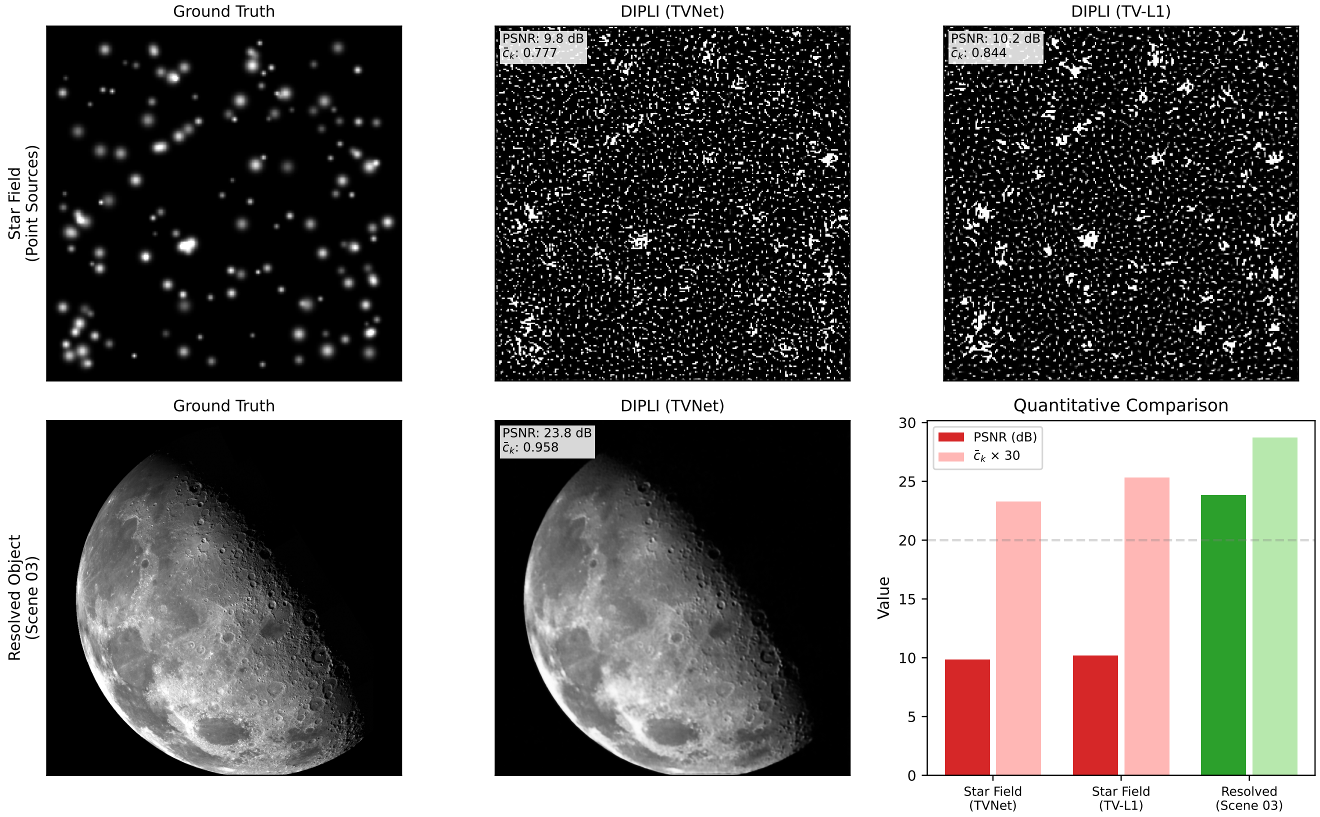

Supplementary Figure 5: Failure case analysis. **Top row:** Star field (point sources); DIPLI fails catastrophically regardless of the flow method (TVNet or TV-L1). **Bottom row:** Resolved Scene 03 (Moon surface) reconstructs successfully under identical hyperparameters. The bar chart compares PSNR and mean confidence across configurations.

The star field experiment reveals two complementary failure mechanisms:

1. **Flow estimation failure.** Mean confidence drops sharply for the star field ($\bar{c}_k = 0.78$ with TVNet, 0.84 with TV-L1) relative to the resolved scene (0.96). Point sources lack the spatial texture gradients that dense optical flow methods rely on. Both TVNet and TV-L1 employ a variational smoothness prior (total variation regularization) that interpolates across the large textureless gaps between point sources, producing unreliable flow vectors in those regions.

2. **Prior mismatch.** The U-Net's implicit prior favors smooth, locally correlated structures, making it fundamentally mismatched to the sparse, discontinuous intensity distribution of a point-source field.

Configuration	$\bar{c}_k$	PSNR	SSIM	LPIPS	DISTS
Star field (TVNet)	0.777	9.84	0.004	0.620	0.460
Star field (TV-L1)	0.844	10.19	0.001	0.591	0.463
Resolved, Scene 03 (TVNet)	0.958	23.83	0.326	0.097	0.074

Supplementary Table 4: Failure case analysis: synthetic star field (point sources) vs. resolved object (Scene 03).

5 Supplementary Note 5: Real Astronomical Data: ZTF Reconstructions

To extend DIPLI's validation beyond the 15 real-data scenes presented in the main text (all resolved solar-system objects), we applied it to 6 targets from the Zwicky Transient Facility (ZTF) [3] survey, a fundamentally different data regime consisting of small, noisy cutouts from a wide-field survey telescope.

5.1 Data description

Each ZTF target is a 60×60 pixel grayscale cutout with 11–13 frames available. The targets span diverse morphological types:

- **Crab Nebula**: diffuse supernova remnant with bright embedded stars
- **M31 (Andromeda)**: extended galaxy with central bulge, low surface brightness
- **NGC 2403**: face-on spiral galaxy
- **M13**: globular cluster (point-source dominated; expected failure case)
- **Two generic ZTF survey fields**: mixed morphology

We used $n_{\text{channels}} = 64$ (reduced from 128 to prevent overfitting on the smaller 60×60 inputs), $N = 5000$ iterations, $n_0 = 4500$, $\sigma_\xi = 0.0025$, and TVNet flow estimation. The SGLD noise scale and learning rate were kept identical to the main experiments; only the architecture capacity and iteration count were adapted to the smaller spatial extent. Because no ground truth is available for these targets, we report no-reference metrics only.

5.2 Results and discussion

Supplementary Table 5: ZTF real-data reconstruction: no-reference metrics. Targets ordered by mean confidence.

Target	K	$\bar{c}_k$	Laplace ↑	BRISQUE ↓	Morphology
ZTF J174+55	13	0.773	0.157	149.0	Dense field
49p864+41p635	12	0.947	0.189	73.9	Spiral galaxy
Crab Nebula	11	0.947	0.211	61.4	Diffuse nebula
M13	12	0.949	1.346	66.1	Globular cluster
M31	11	0.975	0.098	105.9	Extended galaxy
NGC 2403	11	0.986	4.115	88.8	Spiral galaxy

The ZTF experiments extend DIPLI's demonstrated applicability in several important ways:

1. **Diffuse and extended targets succeed.** The Crab Nebula (Supplementary Figure 6, top left) shows clear enhancement of nebular filamentary structure and embedded stars. The spiral galaxy at coordinates J049+41 (row 2, left) exhibits strikingly enhanced spiral arm detail. NGC 2403 achieves the highest confidence ($\bar{c}_k = 0.986$) and sharpness (Laplace = 4.12).
2. **Low-surface-brightness objects are viable.** M31 ($\bar{c}_k = 0.975$) produces a clear reconstruction of the central bulge, despite the potential difficulty posed by its low-surface-brightness outer regions.

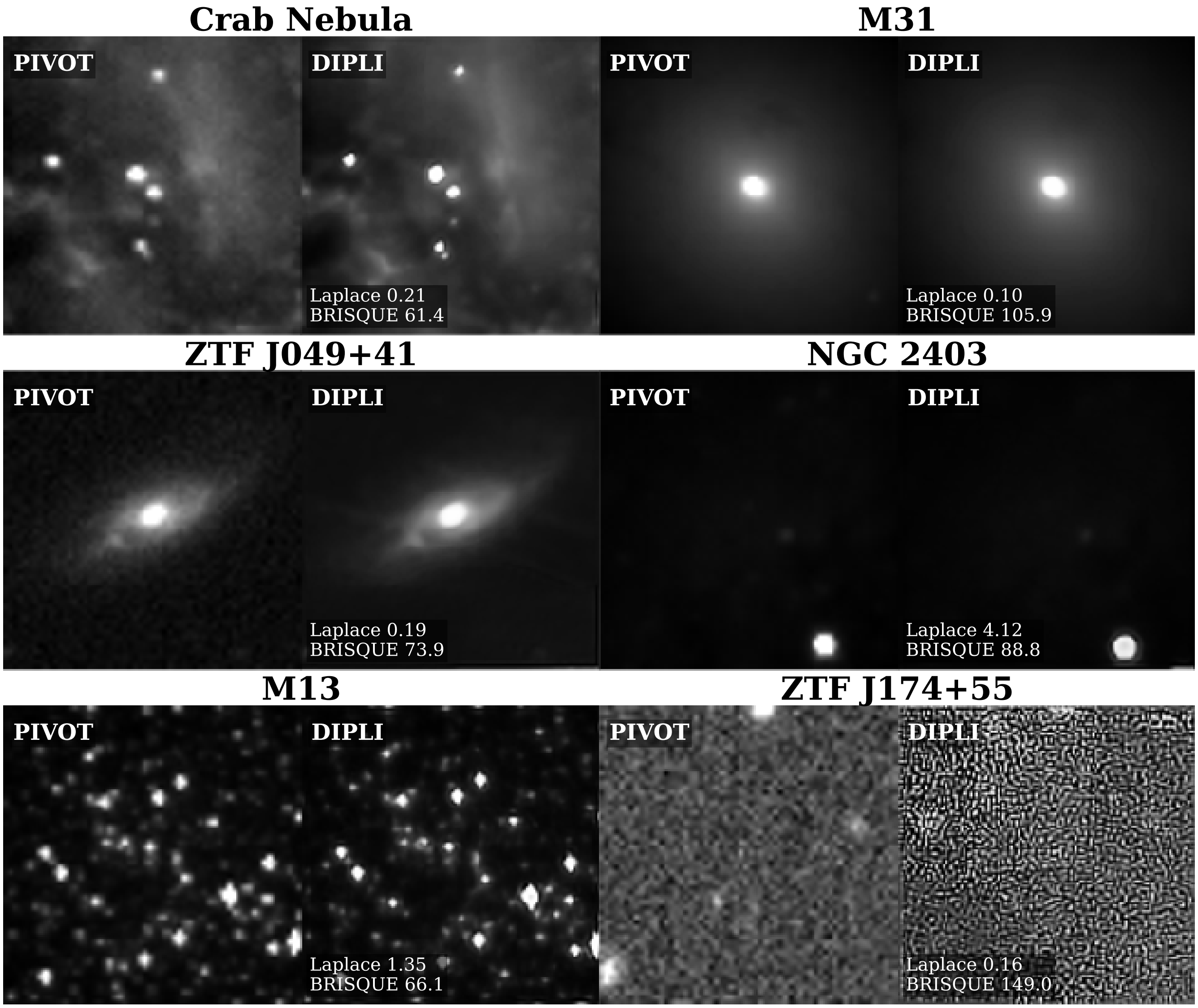

Supplementary Figure 6: **ZTF reconstruction gallery.** For each target: the best input frame (PIVOT, left) and the DIPLI $\times 2$ reconstruction (right). Laplacian energy and BRISQUE are reported for each reconstruction; mean confidence $\bar{c}_k$ is annotated in green (> 0.9) or red (< 0.9). DIPLI successfully enhances resolved and diffuse targets (rows 1–2) but produces characteristic DIP texture artifacts on the dense stellar field J174+55 (bottom right, $\bar{c}_k = 0.77$), consistent with the synthetic failure case (Supplementary Note 4).

3. **Dense and crowded fields struggle.** ZTF J174+55 ($\bar{c}_k = 0.773$, BRISQUE = 149) exhibits characteristic DIP texture artifacts, consistent with the synthetic star field failure case (Supplementary Note 4).

4. **M13 illustrates a boundary case.** The globular cluster ($\bar{c}_k = 0.949$) achieves high confidence because its inter-frame displacements are small and uniform (the cluster fills the frame). However, the unusually high Laplacian energy (1.346) indicates that unresolved point sources are being sharpened rather than truly resolved; the reconstruction enhances individual stellar profiles without separating them. This case illustrates the boundary between the "resolved object" and "point-source field" regimes.

5. **Confidence as a diagnostic.** The mean confidence $\bar{c}_k$ reliably separates successful reconstructions (> 0.9) from the failing dense field (0.773), confirming its utility as a quality indicator.

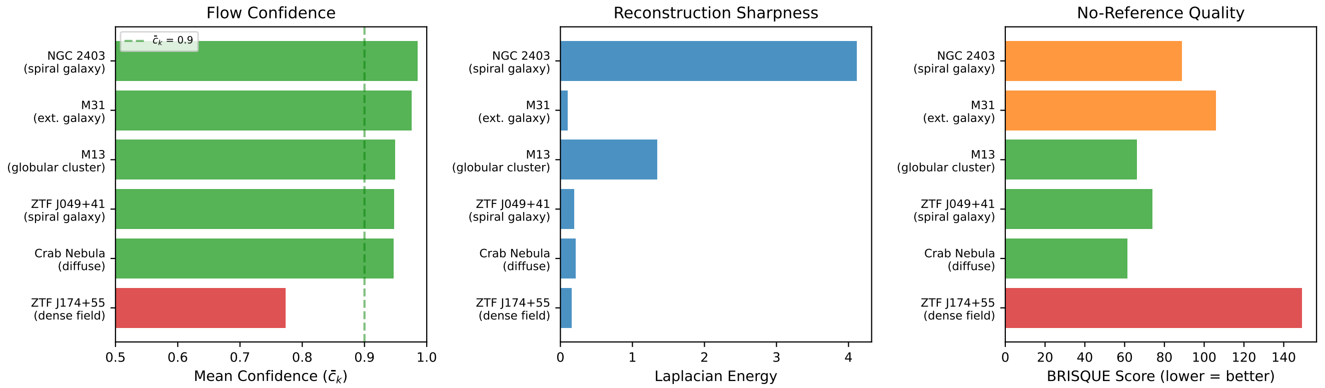

Supplementary Figure 7: No-reference quality metrics for all 6 ZTF targets. **Left:** Mean flow confidence (green: reliable, red: degraded). **Center:** Laplacian energy (sharpness). **Right:** BRISQUE score (lower is better, though calibrated for natural images).

Computational Cost

As reported in Section 4 of the main text, the full DIPLI pipeline (TVNet flow estimation, SGLD optimization for $N = 6500$ iterations, and Monte Carlo averaging) completes in approximately 3–5 minutes per 256×256 scene on a single NVIDIA V100 GPU. The dominant cost is the iterative optimization loop; TVNet flow estimation is a one-time preprocessing step that adds less than 10% overhead.

For the smaller ZTF inputs (60×60 pixels, $N = 5000$, $n_{\text{channels}} = 64$; see Supplementary Note 5), processing completes in under 1 minute per target on the same hardware. Memory usage scales primarily with the number of feature channels and the spatial resolution: the 128-channel configuration used for 256×256 inputs requires approximately 4 GB of GPU memory, well within the capacity of modern consumer GPUs.

Supplementary References

References

[1] Gelman, A. & Rubin, D. B. Inference from iterative simulation using multiple sequences. *Stat. Sci.* **7**, 457–472 (1992).

[2] Blau, Y. & Michaeli, T. The perception-distortion tradeoff. In *Proc. IEEE Conf. Comput. Vis. Pattern Recognit. (CVPR)*, 6228–6237 (2018).

[3] Bellm, E. C. et al. The Zwicky Transient Facility: system overview, performance, and first results. *Publ. Astron. Soc. Pac.* **131**, 018002 (2019).

[4] Cheng, Z., Gadelha, M., Maji, S. & Sheldon, D. A Bayesian perspective on the Deep Image Prior. In *Proc. IEEE Conf. Comput. Vis. Pattern Recognit. (CVPR)*, 5438–5446 (2019).
